# Factors Associated with Tobacco Smoking and Cessation among HIV-Infected Individuals under Care in Rio de Janeiro, Brazil

**DOI:** 10.1371/journal.pone.0115900

**Published:** 2014-12-23

**Authors:** Thiago S. Torres, Paula M. Luz, Monica Derrico, Luciane Velasque, Eduarda Grinsztejn, Valdiléa G. Veloso, Sandra W. Cardoso, Marília Santini-Oliveira, Beatriz Grinsztejn, Raquel Brandini De Boni

**Affiliations:** 1 Instituto de Pesquisa Clínica Evandro Chagas, Fundação Oswaldo Cruz, HIV/AIDS Clinical Research Center, Rio de Janeiro, Brazil; 2 Departamento de Matemática, Universidade Federal do Estado do Rio de Janeiro (UniRio), Brazil; University of Missouri-Kansas City, United States of America

## Abstract

Worldwide the prevalence of smoking among people living with HIV/AIDS is elevated compared to the general population. This probably reflects the cluster of individual characteristics that have shared risk factors for HIV infection and smoking. A cross-sectional study, enrolling a convenience sample from a Brazilian HIV clinical cohort was conducted to evaluate the prevalence of tobacco smoking and the factors associated with current smoking and abstinence. A total of 2,775 HIV-infected individuals were interviewed: 46.2% have never smoked, 29.9% were current smokers and 23.9% were former smokers. Current smokers had a higher prevalence of alcohol and illicit drug use when compared to the other two groups. A higher proportion of heterosexual individuals were former smokers or never smokers while among men who have sex with men (MSM) a higher proportion were current smokers. Former smokers had been more frequently diagnosed with high blood pressure, diabetes mellitus, cardiovascular diseases and depression, while for current smokers lung diseases were more frequent. Former smokers and current smokers were more likely to have had any hospital admission (42.0% and 41.2%, respectively) than participants who never smoked (33.5%) (p<0.001). Multivariate model results showed that current smokers (versus never smokers) were more likely to be less educated, to report the use of alcohol, crack and cocaine and to present clinical comorbidities. Former smokers (versus current smokers) were more likely to be older, to have smoked for a shorter amount of time and to have smoked >31 cigarettes/day. MSM (compared to heterosexuals) and cocaine users (versus non-users) had lower odds of being former smokers. Considering our results, smoking cessation interventions should be tailored to younger individuals, MSM and substance users.

## Introduction

The prevalence of smoking among people living with HIV/AIDS is elevated compared to the general population in both high income countries [1]–[6] and low or middle income countries (LMIC) [7]–[11], although up to 70% of smokers live in LMIC [12]. This probably reflects the cluster of individual characteristics that have shared risk factors for HIV infection and smoking (e.g. younger age, low education level, low socioeconomic status, illicit drug and alcohol use), rather than being a causal relationship [6], [13], [14].

Smoking is a well-established risk factor for several comorbid conditions among HIV-infected individuals, including: pulmonary infectious diseases, non AIDS-defining cancers (e.g. lung cancer), cardiovascular diseases (CVD) and tuberculosis [15]–[23]. Some authors have highlighted significant association between nicotine dependence, depression and combined antiretroviral therapy (cART) poor-adherence [11], [24], [25], but it increases all-cause mortality, even after controlling for CD4+ T-cell count and HIV viral load [5], [15]–[23], [26]–[28]. Additionally, it may limit the effectiveness of cART by promoting HIV-1 gene expression [29]. Recent findings suggest that HIV-infected smokers report a desire to quit but may have substantial difficulties in the process. It is believed that these difficulties may be strengthened by comorbid psychiatric conditions and social support networks comprised mainly by other smokers [30]–[33].

In Brazil, after several regulatory policies, the prevalence of smoking in the general population has declined and it is estimated at 14.8% among the general population [34]. However, very limited data is available on the prevalence of smoking among individual living with HIV/AIDS in the country [7]. The understanding of factors associated with smoking and smoking cessation is crucial to plan targeted interventions for these individuals. The aim of this study is to describe the prevalence of tobacco smoking as well as the factors associated with current smoking and abstinence in a sample of HIV-infected individuals under care at the Instituto de Pesquisa Clínica Evandro Chagas/FIOCRUZ (IPEC) clinical cohort.

## Materials and Methods

This was a cross-sectional study that enrolled a convenience sample selected within the population of the IPEC/FIOCRUZ clinical cohort that had at least one clinical appointment between January 01, 2011 and July 31, 2013.

**Figure 1:**
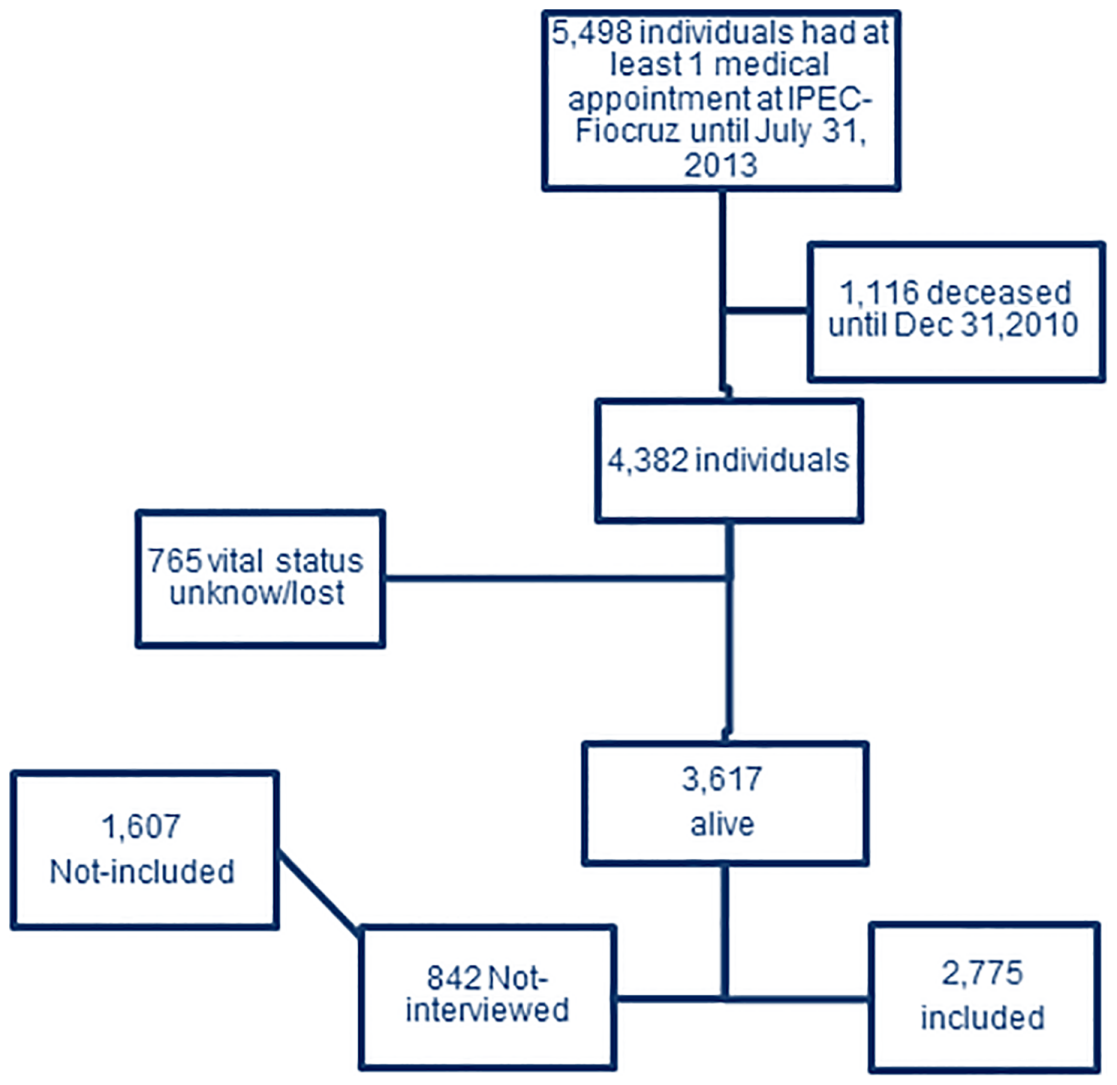
Study Population.

Details of the cohort procedures, definitions and results have been described in previous publications [35]–[37]. Between 1986 and July 31, 2013, 5,498 HIV-infected individuals had at least one clinical appointment at IPEC. Among those, 1,116 were deceased and 765 had unknown vital status or were lost by December 31, 2010. In total, 3,617 patients were alive and potentially eligible for the cross-sectional interview that started in January 01, 2011 (Fig. 1).

### Participants

To be included in the cohort a participant must be HIV-infected, 18 years of age or older, and must have signed an informed consent. For this cross-sectional study, cohort participants were invited to participate either when attending a routine appointment or were reached by phone using a structured phone interview.

### Measures and definitions

Trained nurses administered standardized questionnaire assessing tobacco consumption in interviews that took place in the patients' regular clinical appointments or by phone calls. The questionnaire was comprised by the following questions: 1) Have you ever smoked cigarettes or any other tobacco products in any moment of your life? (If No, stop the questionnaire; if Yes: age of smoking initiation in years); 2) Do you currently smoke cigarette or any other tobacco product? (Yes or No: If No, age when quit smoking in years); and, 3) How many cigarettes do you currently smoke or used to smoke per day?”. Individuals were grouped as 1) never smoked; 2) current smoker (smoking or in abstinence for less than twelve months) and 3) former smokers (cessation for more than twelve months).The mean number of smoked cigarettes/day was categorized into less than 10; 11–20; 21–30 and 31 or more [38].

Data collected during these interviews were linked with the cohort clinical database using the identification number attributed to each patient. This observational, longitudinal, clinical database was established in 1998 (when all patients seen from 1986 to 1998 were retrospectively included) and is updated for all patients receiving HIV and specialty care (i.e. cardiology, endocrinology, ophthalmology, dermatology, gastroenterology, gynecology and proctology) at the clinic. It is a comprehensive database containing demographic, clinical, and therapeutic information abstracted from the medical records of patients. Prescription of antiretrovirals (drug, dosage, and usage) is documented by the medical provider and support staff in the medical records. Trained abstractors record this information onto standardized forms for processing with regular updates to the database.

Data obtained from the clinical database included socio-demographic, behavioral, clinical and therapeutic information, as follows:

- *Race/ethnicity*: self-reported and categorized into white and non-white.
- *Education*: categorized according to the number of years of schooling into lower or equal/higher than 9 years (equivalent to lower and higher than high school).
- *Heavy alcohol use*: patient's lifetime self-report ingestion of more than 20 drinks/week (men) or 14 drinks/week (women).

*Heavy cocaine or crack use* was defined based on a patients' lifetime self-report of weekly to daily use of snorted or smoked cocaine.

*HIV exposition category was categorized* as heterosexual, men who have sex with men (MSM), injection drug use (IDU) and other/unknown.

*Comorbidities were defined as* clinical diagnosis registered in patient's chart during cohort follow-up. High blood pressure, diabetes (DM), chronic obstructive pulmonary disease (COPD), tuberculosis, pneumonia and non-AIDS-related cancer were evaluated. Cardiovascular disease (CVD) was considered as cardiac arrhythmia, heart failure, coronary disease, ischemic heart disease, peripheral vascular insufficiency and stroke. A positive report of treatment for depression was considered as a proxy for depression diagnosis.

*Hospital admission* was defined as any hospitalization up to questionnaire administration.

*Nadir CD4 cell count was* defined as the lowest CD4^+^ T cell count (cells/mm^3^) measured during follow-up, categorized in lower or equal/higher than 200 cells/mm^3^.

Combined antiretroviral treatment (cART) was defined as the use of at least three antiretroviral drugs: two nucleoside reverse transcriptase inhibitor (NRTI) and either one protease inhibitor (PI) or one non-nucleoside reverse transcriptase inhibitor (NNRTI), and categorized into ever and never used cART.

### Data analysis

Descriptive analysis and proportions of current smokers, former smokers and never smokers were performed. Chi-square tests and Fisher exact tests were used for categorical variables, and Kruskal-Wallis was used for the asymmetric continuous variable.

Univariate analyses were conducted to compare 1) never smokers with current smokers and; 2) current smokers with former smokers. Two logistic regression models were used to identify independent variables associated with current smoking and smoking abstinence. After univariate analysis, covariates with p-values <0.20 were selected and entered in the initial multivariate model. Covariates with the highest p-values in the analysis were sequentially removed. Variables with statistical significance at 5% (p<0.05) and those that were considered as confounders (e.g., when removed, a change equal to or higher than 10% in the odds ratio of any other variable of the model was observed) remained in the final model. The softwares R 3.0.2 (R Foundation for Statistical Computing: R. 2013) and IBM SPSS version 20 were used to generate all the analyses.

### Ethics

IPEC-FIOCRUZ institutional review board has approved this study and all study participants have signed an informed consent form.

### Results

A total of 2,775 (76.7%) of individuals under active follow-up responded the questionnaire; 1,281 have never smoked (46.2%), 830 (29.9%) were current smokers and 664 (23.9%) were former smokers.

Table 1 shows the characteristics of the sample stratified by smoking status. The majority of participants were male (65.4%) and the overall male-female ratio was 1.89∶1. This ratio is similar for former smokers and those who never smoked (1.67∶1 and 1.61∶1) but higher for current smokers (2.70∶1) (p<0.001). Former smokers were on average 5 years older than the other two groups.

**Table 1:** Demographic and clinical characteristics stratified by smoking status, HIV-infected individuals under care at IPEC, 2011–13.

	Current smoker 830(29.9%)	Former smoker 664(23.9%)	Never smoked 1281(46.2%)	p-value*
Male	606(73.0)	416(62.5)	792(61.8)	<0.001
Age				<0.001
<30 years	161(19.4)	77(11.6)	224(17.5)	
31–40 years	249(30.0)	132(19.9)	463(36.1)	
41–50 years	257(31.0)	240(36.1)	391(30.5)	
51 years or more	163(19.6)	215(32.4)	203(15.8)	
Schooling				
Less than 9 nine years	440(53.1)	329(49.7)	580(45.4)	0.002
9 years or more	388(46.9)	333(50.3)	698(54.6)	
White	447(54.1)	370(55.8)	659(51.7)	0.21
Heavy alcohol use	137(16.5)	74(11.1)	92(7.2)	<0.001
Cocaine inhaled	162(19.5)	47(7.1)	67(5.2)	<0.001
Crack use	28(3.4)	5(0.8)	2(0.2)	<0.001 **
Time of tobacco use in years(min-max))	23(0–67)	12(0–52)	-	<0.001***
Cigarettes per day			-	<0.001
<10	415(50.4)	380(57.7)	-	
11–20	305(37.1)	171(25.9)	-	
21–30	37(4.5)	14(2.1)	-	
31 or more	66(8.0)	94(14.3)	-	
HIV exposure category				<0.001
Heterosexuals	349(42.0)	360(54.2)	681(53.2)	
MSM	278(33.5)	156(23.5)	319(24.9)	
IDU	23(2.8)	11(1.7)	9(0.7)	
Other/unknown	180(21.7)	137(20.6)	272(21.2)	
Clinical comorbidities				
High blood pressure	208(25.1)	249(37.5)	338(26.4)	<0.001
DM	79(9.5)	106(16.0)	147(11.5)	0.001
CVD	61(7.3)	63(9.5)	54(4.2)	<0.001
COPD	80(9.6)	54(8.1)	65(5.1)	<0.001
TB	249(29.9)	160(24.1)	256(20.0)	<0.001
Pneumonia	248(30.0)	198(29.8)	288(22.5)	<0.001
Non-AIDS defining cancer	18(2.2)	25(3.8)	45(3.5)	0.12
Depression treatment	243(29.3)	203(30.6)	295(23.0)	0.001
Hospital admission	342(41.2)	279(42.0)	429(33.5)	<0.001
cART	757(91.2)	630(94.9)	1161(90.6)	0.004
Nadir CD4^+^ T cell (cells/mm^3^)				0.364
< = 200	434(52.4)	365(55.0)	659(51.6)	
>200	395(47.6)	299(45.0)	618(48.4)	

*Chi-square,

**Fischer exact test,

***Kruskal-Wallis.

Current smokers had a higher prevalence of alcohol and illicit drug use when compared to the other two groups. When compared to former smokers, current smokers had a longer time of tobacco use (12 years IQR: 0–52 and 23 years IQR: 0–67, respectively; p<0.001) and were more likely to smoke >10 cigarettes/day (42.3% and 49.6%, respectively).

A higher proportion of heterosexual individuals were former smokers or never smokers while among MSM a higher proportion were current smokers (p<0.001).

Former smokers have been diagnosed more frequently with high blood pressure (37.5%; p<0.001), DM (16.0%; p = 0.001), CVD (9.5%; p<0.001) and depression (30.6%; p = 0.001), while for current smokers COPD (9.6%; p<0.001), TB (29.9%; p<0.001) and pneumonia (30.0%) were more frequent. Former smokers and current smokers were more likely to have any hospital admission (42.0% and 41.2%, respectively) than participants who never smoked (33.5%) (p<0.001).

At the first logistic regression model, individuals who were current smokers, compared to never smokers, were more likely to report the use of crack cocaine (AOR 7.49, CI 95%1.69–33.13), inhaled cocaine (AOR 3.42; IC 95% 2.46–4.75) and alcohol (AOR 1.71; CI 95%1.26–2.34). They were less likely to have more than 9 years of education (AOR 0.69; IC95% 0.57–0.84). Clinical comorbidities as CVD, COPD, TB and depression were independently associated with current smoking, as depicted in Table 2.

**Table 2:** Factors associated with current smoking compared to those who never smoked, HIV-infected patients under care at IPEC, 2011–2013.

	Crude OR (CI95%)	p-value	Adjusted OR(CI95%)
Male	1.67(1.38–2.02)	<0.001	-
Age			
<30 years	1	0.013	1
31–40 years	0.75(0.58–0.96)	0.025	0.69(0.52–0.90)
41–50 years	0.91(0.71–1.18)	0.495	0.82(0.62–1.08)
51 years or more	1.12(0.84–1.49)	0.452	1.08(0.79–1.47)
Schooling		0.002	
Less than 9 nine years	1		1
9 years or more	0.73(0.61–0.87)		0.69(0.57–0.84)
Non-white	0.63(0.16–2.46)	0.484	-
HIV exposition category		0.001	
Heterosexuals	1		1
MSM	1.70(1.38–2.09)		2.36(1.87–2.98)
IDU	4.99(2.28–10.89)		2.89(1.24–6.67)
Other/unknown	1.29(1.03–1.62)		1.37(1.07–1.75)
Heavy alcohol use	2.55(1.93–3.38)	<0.001	1.71(1.26–2.34)
Cocaine inhaled	4.39(3.26–5.93)	<0.001	3.42(2.46–4.75)
Crack use	22.33(5.30–93.98)	<0.001	7.49(1.69–33.13)
Clinical comorbidities			
High blood pressure	0.93(0.76–1.14)	0.496	-
DM	0.81(0.61–1.08)	0.153	-
Cardiovascular disease	1.80(1.24–2.63)	0.002	1.67(1.11–2.50)
COPD	1.99(1.42–2.80)	<0.001	1.99(1.39–2.86)
TB	1.71(1.39–2.09)	<0.001	1.45(1.17–1.81)
Pneumonia	1.48(1.21–1.80)	<0.001	-
cancer	0.61(0.35–1.06)	0.07	0.46(0.25–0.83)
Depression	1.38(1.13–1.69)	<0.001	1.28(1.03–1.59)
Lifetime Hospital admission	1.39(1.16–1.67)	<0.001	-
cART	0.93(0.69–1.27)	0.665	-
Nadir CD4^+^ T cell (cells/mm^3^)		0.737	-
< = 200	1		
>200	0.97(0.81–1.16)		

Variables with p-values <0.20 entered in the initial multivariate model. Those with statistical significance at 5% (p<0.05) or confounders remained in the final model.

Regarding smoking cessation, the second logistic model showed that former smoker were more likely to be older, to have smoked for a shorter period of time and to have smoked >31 cigarettes/day than current smokers. MSM had a lower odds of being former smoker (AOR 0.51; CI 95% 0.36–0.71) compared to heterosexuals. Cocaine users (AOR 0.37; CI 95% 0.23–0.58) also had lower odds of being former smoker when compared to non-users (Table 3).

**Table 3:** Factors associated with smoking cessation (former smokers) compared to current smoking, HIV-infected patients under care at IPEC, 2011–2013.

	Crude OR(CI 95%)	p-value	Adjusted OR(CI95%)
Male	0.62(0.49–0.77)	<0.001	-
Age			
<30 years	1		1
31–40 years	1.11(0.79–1.56)	0.557	3.73(2.39–5.81)
41–50 years	1.95(1.41–2.7)	<0.001	33.8(19.7–57.9)
51 years or more	2.76(1.96–3.87)	<0.001	351.7(163.5–756.6)
Schooling		0.186	-
Less than 9 nine years	1		-
9 years or more	1.15(0.93–1.41)		-
Non-White	0.40(0.04–3.88)	0.585**	-
Smoking time (years)	0.93(0.92–0.94)	<0.001***	0.8(0.78–0.82)
Cigarettes per day			
<10	1		1
11–20	0.61(0.48–0.77)	<0.001	1.17(0.78–1.62)
21–30	0.41(0.22–0.48)	0.006	0.78(0.32–1.86)
31 or more	1.55(1.01–2.19)	0.012	4.23(2.61–6.87)
HIV exposition category		<0.001	
Heterosexuals	1		1
MSM	0.54(0.43–0.69)		0.51(0.36–0.71)
IDU	0.46(0.22–0.96)		1.69(0.60–4.07)
Other/unknown	0.74(0.56–0.96)		0.61(0.41–0.89)
Heavy alcohol use	1.58(1.16–2.13)	0.003	-
Cocaine inhaled	0.31(0.22–0.44)	<0.001	0.37(0.23–0.58)
Crack use	0.22(0.08–0.57)	<0.001	-
Clinical comorbidities			
High blood pressure	1.79(1.44–2.24)	<0.001	1.84(1.30–2.59)
DM	1.81(1.32–2.46)	<0.001	1.57(0.98–2.51)
CVD	1.32(0.91–1.91)	0.138	-
COPD	0.83(0.58–1.19)	0.310	-
TB	0.74(0.59–0.94)	0.012	-
Pneumonia	0.99(0.79–1.24)	0.940	-
cancer	1.76(0.95–3.26)	0.068	-
Depression treatment	1.06(0.85–1.33)	0.587	-
Lifetime Hospital admission	1.03(0.84–1.27)	0.751	-
cART	1.79(1.17–2.72)	0.005	-
Nadir CD4^+^ T cell (cells/mm^3^)		0.313	-
< = 200	1		-
>200	0.9(0.73–1.11)		-

**Fischer exact test; ***Kruskal-Wallis. Variables with p-values <0.20 entered in the initial multivariate model. Those with statistical significance at 5% (p<0.05) or confounders remained in the final model.

## Discussion

Our study provides insight on tobacco smoking use and factors associated with current smoking and abstinence in a sample selected from a large clinical cohort in Rio de Janeiro, Brazil. The observed prevalence of current smoking (29.9%) was similar to another HIV cohort from Recife, Brazil (28.9%) [7], so far the only information available in the country. Compared to international HIV-cohorts from high [1], [13], [39]–[41] and low [11], [17], [42], [43] income settings (prevalence ranging from 40–67% and 46–47%, respectively), the prevalence found herein was considerably lower. It was also lower than the prevalence found at the SMART clinical trial (40.5%), which included 5472 HIV-infected patients from 33 countries [5]. This overall lower prevalence of current smokers might be reflecting the successful public policies against tobacco adopted during the last decades in Brazil that led to an overall decrease of smoking in the Brazilian population [34].

Nevertheless, as reported in other settings [11], [13], [26], [44], the smoking prevalence found for this study population was twice the prevalence of tobacco use among the Brazilian general population (14.8%) [36]. One third of individuals in our study are still smoking and addressing this behavior must be a priority in the care provision package offered to them. Given widespread cART use, mortality due to AIDS-related causes has decreased [45], [46] while the proportion of non-AIDS related causes of death have increased [36]. In fact, a Danish study has shown that among HIV smoking individuals the number of years lost due to tobacco use was twice the number of years lost by HIV. The authors have estimated that life expectancy of a 35 years old HIV-infected individual who smokes is 62.6 years while for a never-smoker HIV-infected individual, it was estimated in 78.9 years [47], pointing out the significant burden posed by smoking in this population.

Our results show that current smokers were mostly men, with lower education level, higher prevalence of alcohol and drug use and higher prevalence of respiratory disease, findings that are in accordance with the international literature [7], [10], [44], [48]. However, there were no significant differences among the three groups regarding non-AIDS related cancers, contrary to previous studies [5]. This result is most likely related to survival bias, since cancer is a well-known cause of death among HIV-infected individuals [49]. Also, differently from other studies [5], the nadir CD4 was similar among the groups. One could speculate that this lack of difference is related to the late entry in treatment, which is still common in our country [50], [51].

Current smokers, compared to never smokers (Table 2), were more likely to have used other drugs such as alcohol and cocaine and to have less than 9 years of education. The clustering of multiple psychosocial problems among HIV-infected smokers has been described in other studies [6], [30], [31], [44] and may represent a challenge for programs aiming at smoking cessation in this population. Additionally, current smoking was more likely among already stigmatized populations such as MSM [52], [53] and IDU [54], adding challenges to smoking related prevention efforts.

Current smokers had a higher chance of presenting CVD when compared to those who never smoked, which could be expected since tobacco is a well-established risk factor for CVD overall and among the HIV-infected population [15], [19], [20], [55]. Moreover, a Tuberculosis (TB) diagnosis was also more likely for them. This is extremely important in our settings because TB is the main cause of death for HIV-infected individuals in Brazil [36] and prevalence of TB in Rio de Janeiro city is high [56]. Tobacco use was already associated with mortality in tuberculosis patients [57]–[59], as well as a predictor for non-adherence to anti-TB drugs [60], but the impact of interactions between smoking and these diseases on the mortality of HIV-infected individuals under care in Brazil must be better understood in future studies.

Considering the overall individuals who had ever smoked (n = 1,492), 44.5% (664/1492) have succeed on quitting at some point in their lives. Former smokers were more likely than current smokers to be older maybe because most smokers who achieve abstinence need many tries before definitely quitting [61], [62]. They were also more likely to have smoked for a shorter period of time, which may be related to a less severe dependence [63], although a higher number of cigarettes per day was also associated with abstinence. The same psychosocial variables associated to current smoking (being an MSM and using cocaine), were barriers to smoking cessation, as also described by Shirley et al [33]. These findings reinforce the need of targeting interventions to young people and MSM, as well as the need to address the abuse/dependence of other substances. The appropriate interventions, however, are not well defined yet. A growing number of studies are being conducted to address smoking among HIV-infected individuals [64]–[67], but results are still preliminary and larger clinical trials are warranted in this area.

Our unadjusted results also showed that former smokers were more likely to be using cART than current smokers and those who never smoked (Table 1). We believe one possible reason for this finding is the higher prevalence of older patients (>40 years, 68.5%) among former smokers, since the association of smoking with cART was no longer present in the adjusted logistic model (Table 3). A previous analysis of the cohort has indicated that older patients in follow-up were infected while younger and are aging with HIV [35]. As a consequence, a higher proportion of them have started to use cART during follow up. Another plausible explanation would be that patients under cART may receive more systematic counseling to quit smoking, as this procedure has been progressively incorporated into good clinical practice for patients with HIV/AIDS.

The prevention of non-communicable diseases among HIV/AIDS patients is a major concern as a larger proportion of individuals with HIV are living longer and facing the double challenge of HIV infection, with its required lifelong treatment, and the increasing burden of chronic non-communicable diseases associated with tobacco use, such as cancer and cardiovascular diseases [68].

This study has noteworthy limitations. First we did not have a standardized instrument to measure the severity of smoking use, which was marginally inferred by the number of cigarettes and length of smoking. Second, as measures were self-reported and no biochemical verification was made, individuals were prone to memory bias, especially the former smokers, and social desirability bias. Memory bias may have overestimated the number of cigarettes smoked among those who quit. Social desirability bias may have underestimated overall prevalence of smoking as it can be understood as an unapproved behavior. Third, the findings cannot be generalized to entire IPEC clinical cohort due to differences among population enrolled during the 25 years of follow –up, the effect of cART over mortality, possible cohort effects and the non-probabilistic characteristic of the sample selected for the cross-sectional study (S1 Table). Fourth, although the questions used to capture smoking behavior were simple, we did not capture the information of how many individuals answered them face-to-face or by phone. The impact of the two data collection methods may or may not have influenced participants' answers. In addition, our sample included participants who were and who were not on cART, and cART was not associated to smoking or smoking cessation. Future studies should also evaluate how cART effects – both objective (e.g., viral load suppression) and subjective (e.g. quality of life) - are related to smoking/abstinence among individuals under antiretroviral therapy. Finally, data were collected from HIV-infected patients at routine clinic visits (and by telephone). Smoking rates in this population may differ from that not engaged in care, limiting data generalization.

In conclusion, these results suggest that individuals living with HIV/AIDS in our cohort are vulnerable to tobacco use. Smoking cessation interventions must be urgently incorporated into the package of care provided to the most vulnerable populations such as MSM, the less educated and drug users.

## Supporting Information

S1 Table **Characteristics of cohort individuals who were included vs. not included in the cross-sectional tobacco study.**
(PDF)
