# Supplementary material for: Factors Associated with Tobacco Smoking and Cessation among HIV-Infected Individuals under Care in Rio de Janeiro, Brazil
Source: PLoS One. 2014 Dec 23;9(12):e115900. doi: 10.1371/journal.pone.0115900 (PMC4275249; doi:10.1371/journal.pone.0115900)
Supplement: S1 Table — Characteristics of cohort individuals who were included vs. not included in the cross-sectional tobacco study. (PDF) [file pone.0115900.s001.pdf]

Table S1: Characteristics of cohort individuals who were included vs. not included in the cross-sectional tobacco study.

	**Not –included N=1607**	**Included N=2775**	**p-value***
First appointment after January 1st, 1997	1222(76.0)	2516(90.7)	<0.001
Follow-up time higher than 60days	1318(82.0)	2717(97.9)	<0.001
Male	1115(69.3)	1812(65.3)	0.006
Age	34.7(10.7)	36.1(10.0)	<0.001
White	787(49.0)	1475(53.2)	<0.001
HIV exposure category			<0.001
Heterosexuals	637(39.6)	1389(50.1)	
MSM	444(27.6)	753(27.1)	
IDU	25(1.6)	43(1.6)	
Other/unknown	502(31.2)	589(21.2)	
cART use	958(59.6)	2599(93.7)	<0.001
